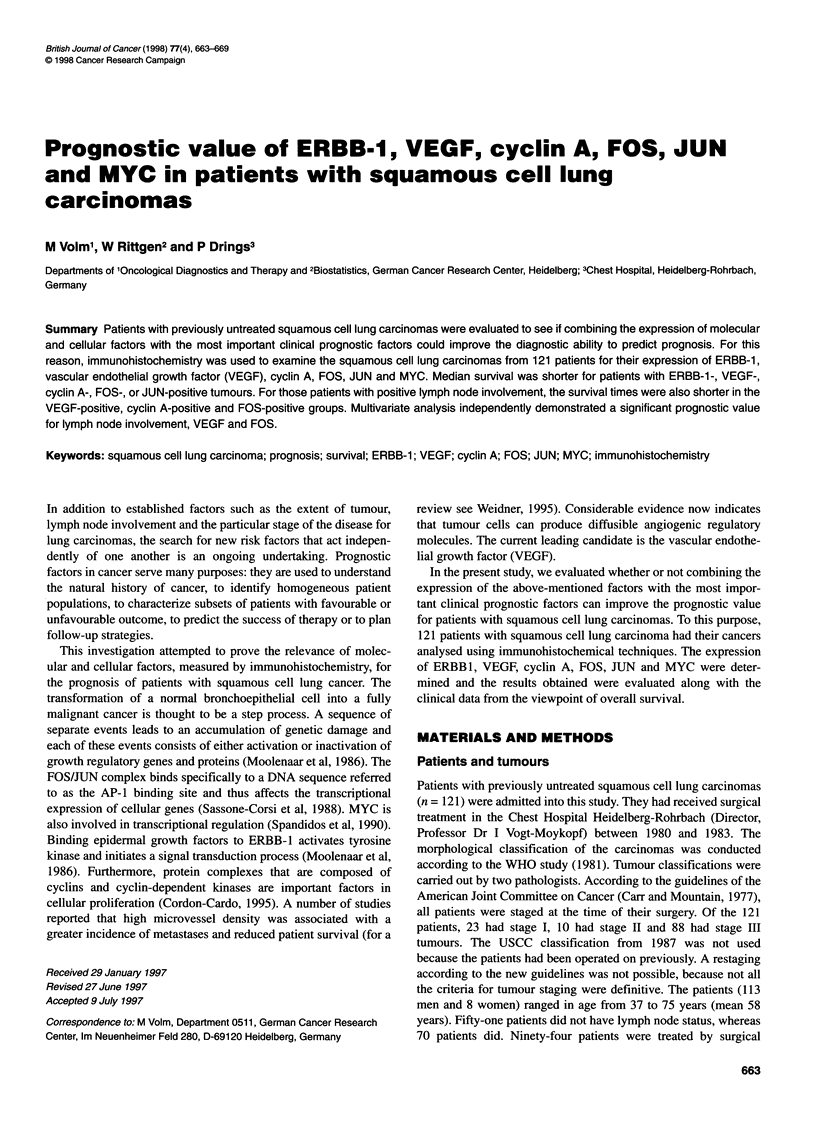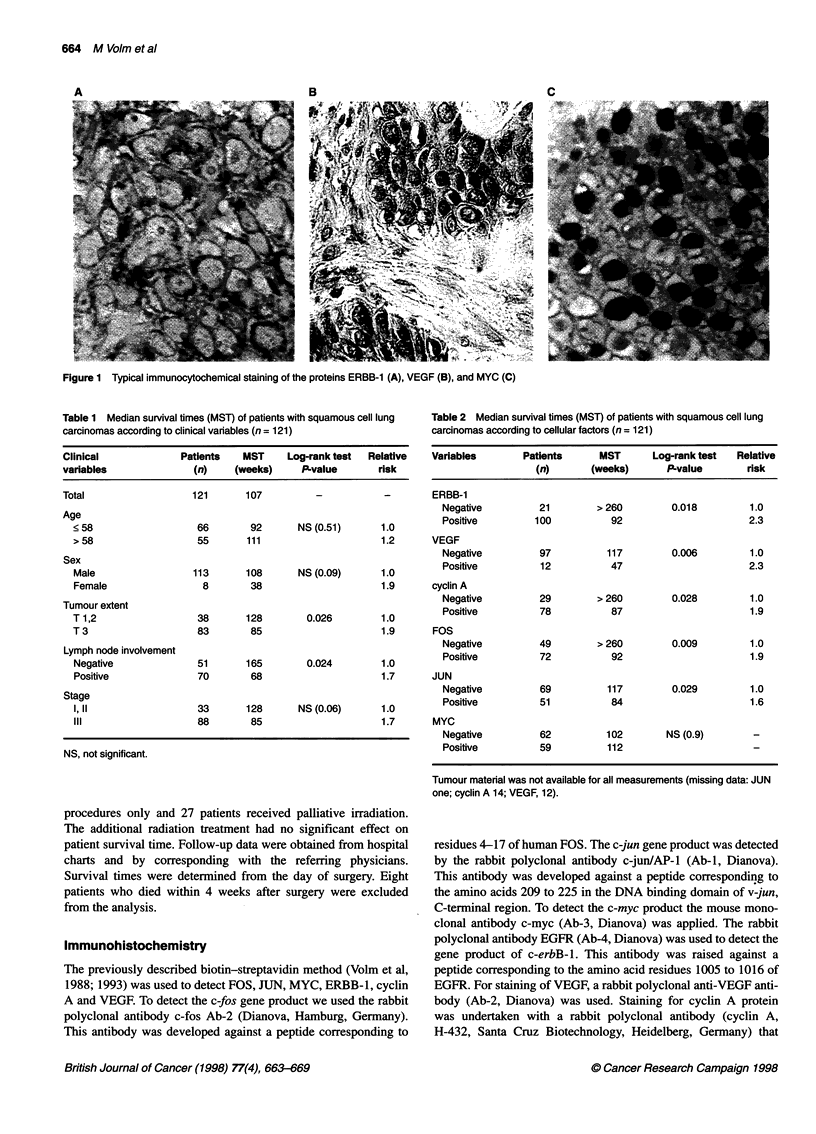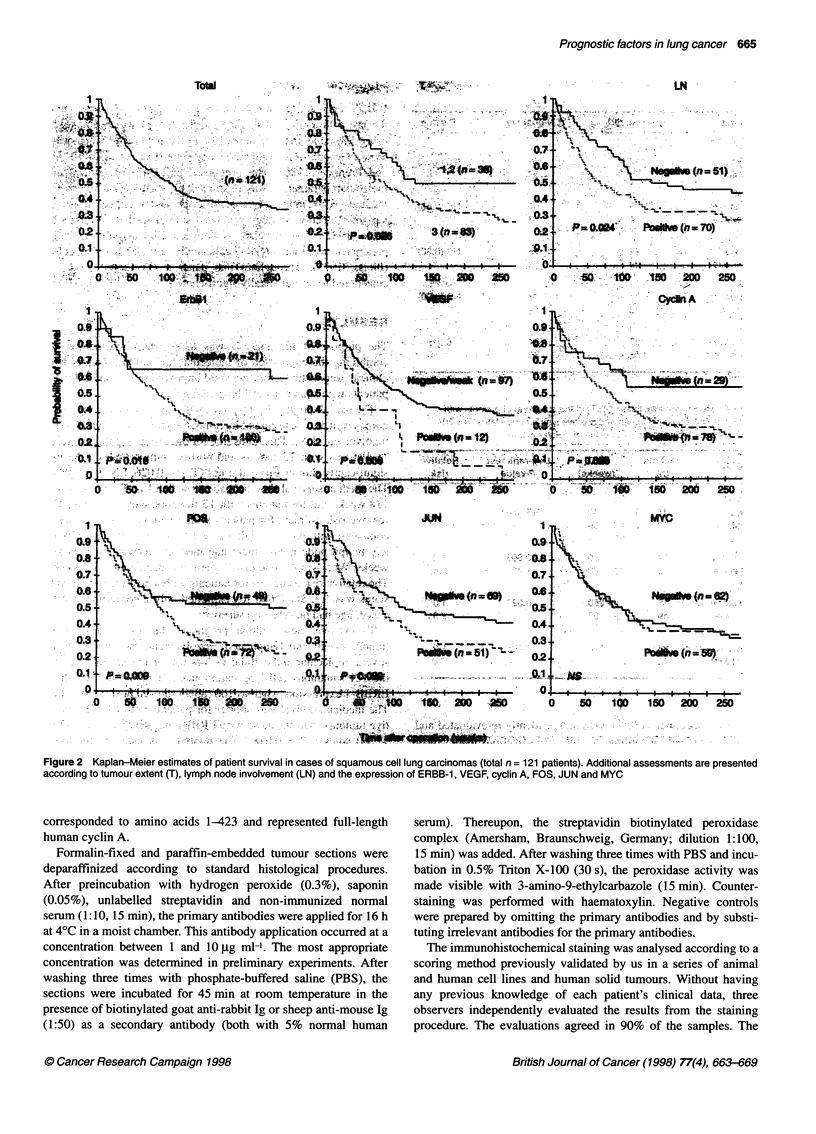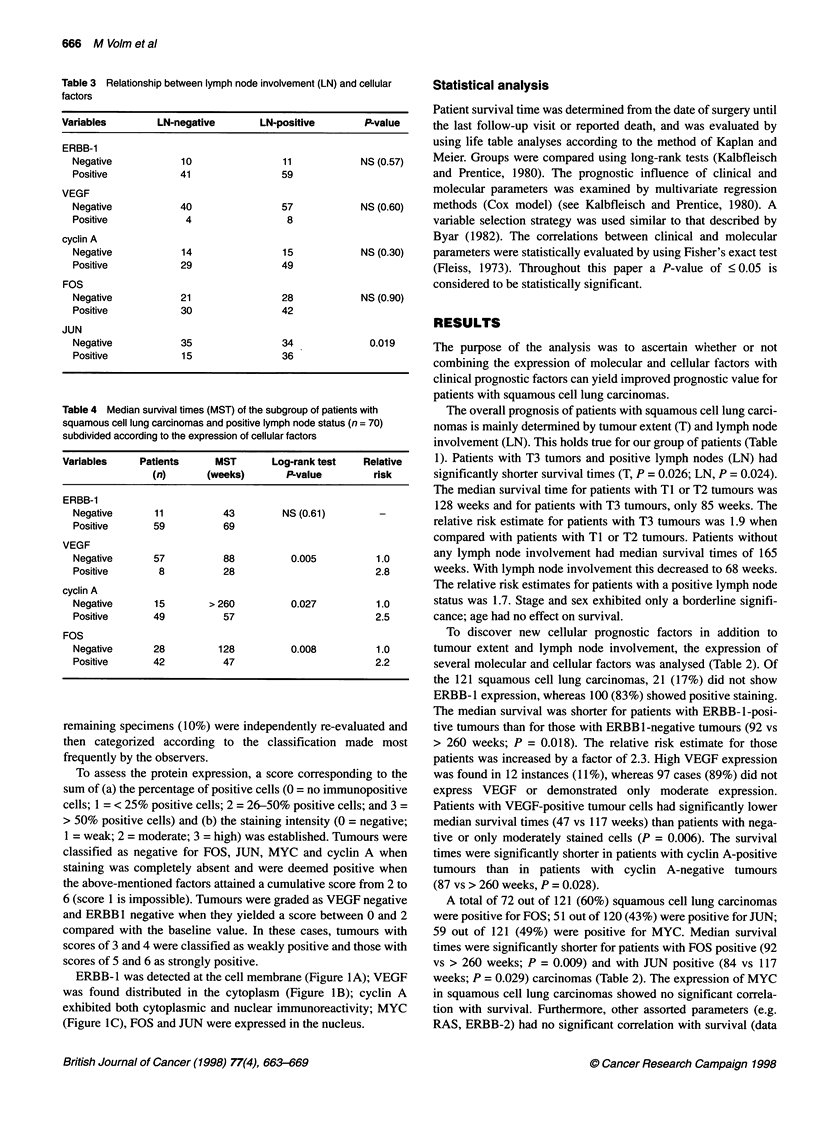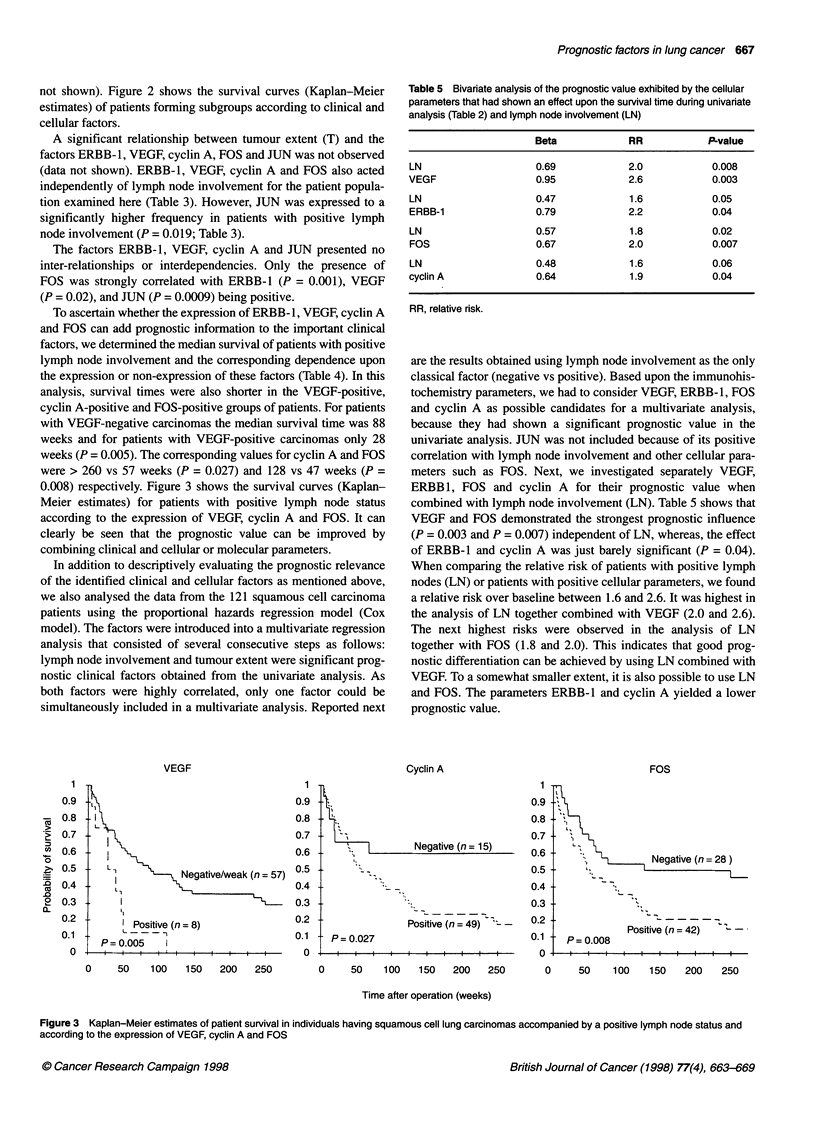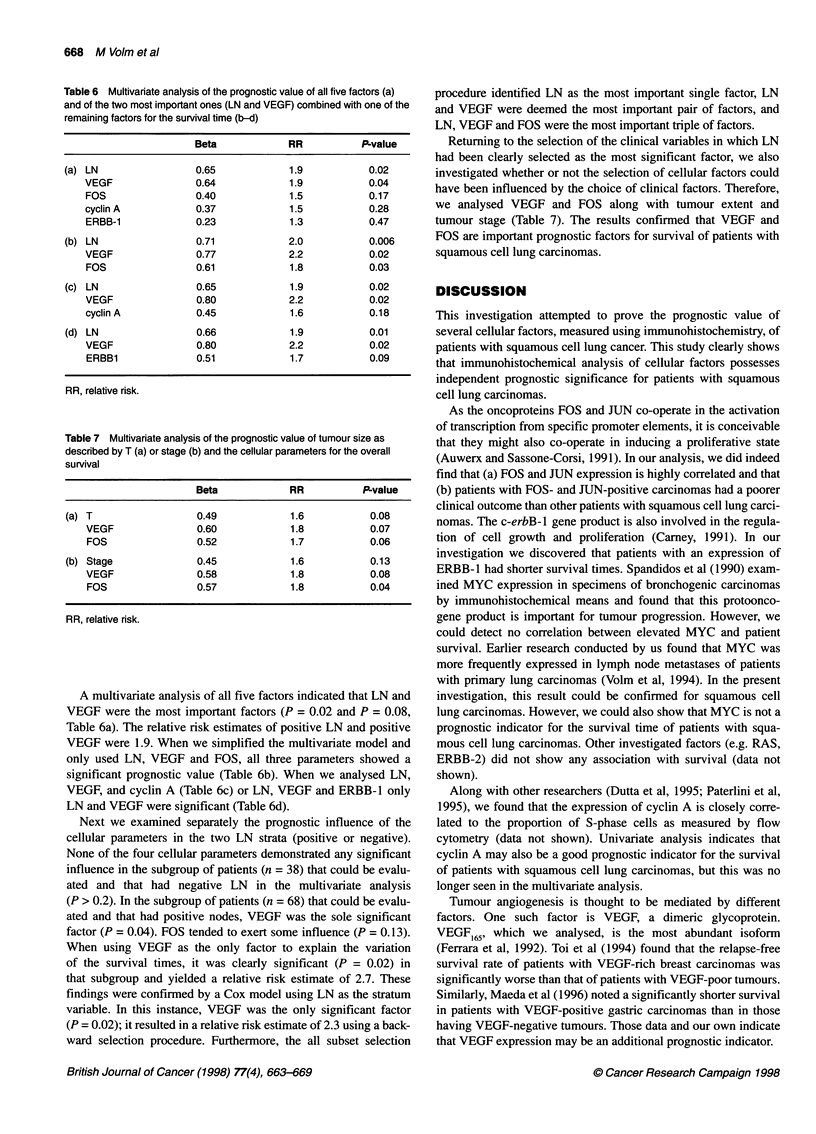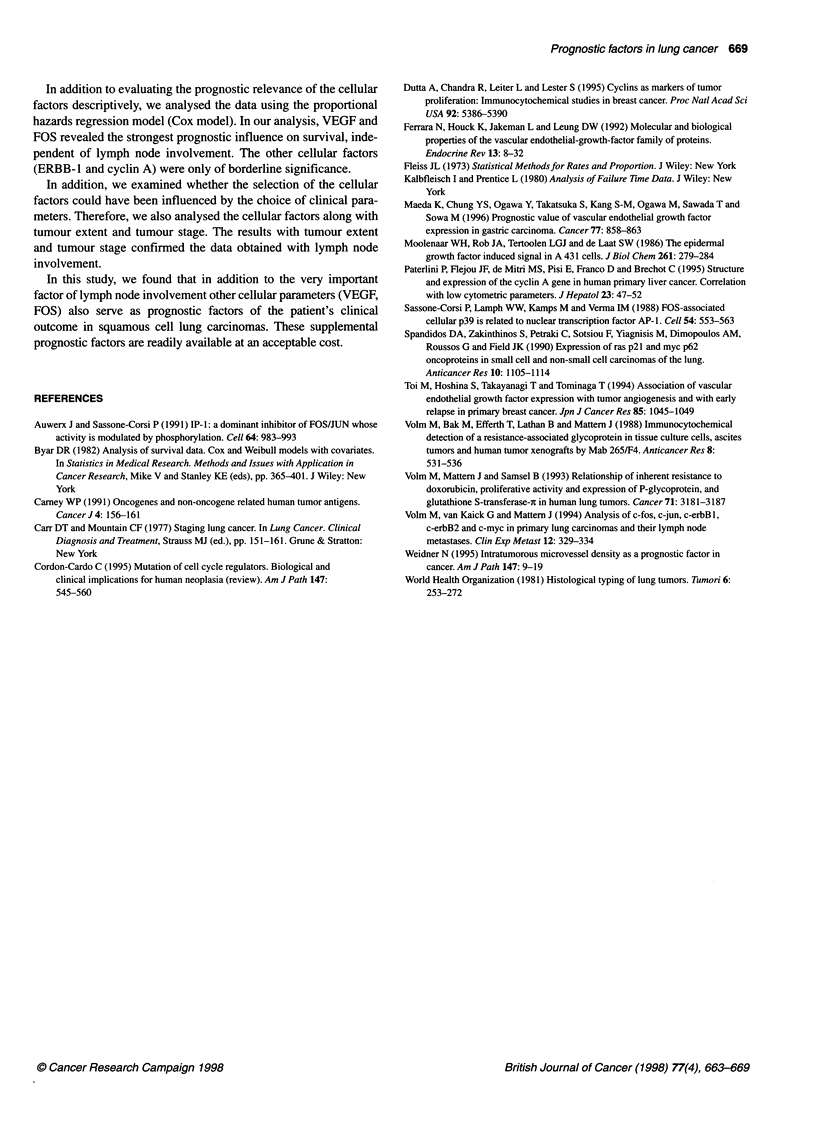# Prognostic value of ERBB-1, VEGF, cyclin A, FOS, JUN and MYC in patients with squamous cell lung carcinomas.

**DOI:** 10.1038/bjc.1998.106

**Published:** 1998-02

**Authors:** M. Volm, W. Rittgen, P. Drings

**Affiliations:** Department of Oncological Diagnostics and Therapy, German Cancer Research Center, Heidelberg.

## Abstract

**Images:**


British Journal of Cancer (1998) 77(4), 663–669
© 1998 Cancer Research Campaign

### Prognostic value of ERBB-1, VEGF, cyclin A, FOS, JUN and MYC in patients with squamous cell lung carcinomas

**M Volm[1], W Rittgen[2] and P Drings[3]**

Departments of [1]Oncological Diagnostics and Therapy and [2]Biostatistics, German Cancer Research Center, Heidelberg; [3]Chest Hospital, Heidelberg-Rohrbach, Germany

**Summary** Patients with previously untreated squamous cell lung carcinomas were evaluated to see if combining the expression of molecular and cellular factors with the most important clinical prognostic factors could improve the diagnostic ability to predict prognosis. For this reason, immunohistochemistry was used to examine the squamous cell lung carcinomas from 121 patients for their expression of ERBB-1, vascular endothelial growth factor (VEGF), cyclin A, FOS, JUN and MYC. Median survival was shorter for patients with ERBB-1-, VEGF-, cyclin A-, FOS-, or JUN-positive tumours. For those patients with positive lymph node involvement, the survival times were also shorter in the VEGF-positive, cyclin A-positive and FOS-positive groups. Multivariate analysis independently demonstrated a significant prognostic value for lymph node involvement, VEGF and FOS.

**Keywords:** squamous cell lung carcinoma; prognosis; survival; ERBB-1; VEGF; cyclin A; FOS; JUN; MYC; immunohistochemistry

In addition to established factors such as the extent of tumour, lymph node involvement and the particular stage of the disease for lung carcinomas, the search for new risk factors that act independently of one another is an ongoing undertaking. Prognostic factors in cancer serve many purposes: they are used to understand the natural history of cancer, to identify homogeneous patient populations, to characterize subsets of patients with favourable or unfavourable outcome, to predict the success of therapy or to plan follow-up strategies.

This investigation attempted to prove the relevance of molecular and cellular factors, measured by immunohistochemistry, for the prognosis of patients with squamous cell lung cancer. The transformation of a normal bronchoepithelial cell into a fully malignant cancer is thought to be a step process. A sequence of separate events leads to an accumulation of genetic damage and each of these events consists of either activation or inactivation of growth regulatory genes and proteins (Moolenaar et al, 1986). The FOS/JUN complex binds specifically to a DNA sequence referred to as the AP-1 binding site and thus affects the transcriptional expression of cellular genes (Sassone-Corsi et al, 1988). MYC is also involved in transcriptional regulation (Spandidos et al, 1990). Binding epidermal growth factors to ERBB-1 activates tyrosine kinase and initiates a signal transduction process (Moolenaar et al, 1986). Furthermore, protein complexes that are composed of cyclins and cyclin-dependent kinases are important factors in cellular proliferation (Cordon-Cardo, 1995). A number of studies reported that high microvessel density was associated with a greater incidence of metastases and reduced patient survival (for a review see Weidner, 1995). Considerable evidence now indicates that tumour cells can produce diffusible angiogenic regulatory molecules. The current leading candidate is the vascular endothelial growth factor (VEGF).

In the present study, we evaluated whether or not combining the expression of the above-mentioned factors with the most important clinical prognostic factors can improve the prognostic value for patients with squamous cell lung carcinomas. To this purpose, 121 patients with squamous cell lung carcinoma had their cancers analysed using immunohistochemical techniques. The expression of ERBB1, VEGF, cyclin A, FOS, JUN and MYC were determined and the results obtained were evaluated along with the clinical data from the viewpoint of overall survival.

### MATERIALS AND METHODS

### Patients and tumours

Patients with previously untreated squamous cell lung carcinomas ($n = 121$) were admitted into this study. They had received surgical treatment in the Chest Hospital Heidelberg-Rohrbach (Director, Professor Dr I Vogt-Moykopf) between 1980 and 1983. The morphological classification of the carcinomas was conducted according to the WHO study (1981). Tumour classifications were carried out by two pathologists. According to the guidelines of the American Joint Committee on Cancer (Carr and Mountain, 1977), all patients were staged at the time of their surgery. Of the 121 patients, 23 had stage I, 10 had stage II and 88 had stage III tumours. The USCC classification from 1987 was not used because the patients had been operated on previously. A restaging according to the new guidelines was not possible, because not all the criteria for tumour staging were definitive. The patients (113 men and 8 women) ranged in age from 37 to 75 years (mean 58 years). Fifty-one patients did not have lymph node status, whereas 70 patients did. Ninety-four patients were treated by surgical

*Received 29 January 1997*
*Revised 27 June 1997*
*Accepted 9 July 1997*

*Correspondence to:* M Volm, Department 0511, German Cancer Research Center, Im Neuenheimer Feld 280, D-69120 Heidelberg, Germany

**Figure 1** Typical immunocytochemical staining of the proteins ERBB-1 (**A**), VEGF (**B**), and MYC (**C**)

**Table 1** Median survival times (MST) of patients with squamous cell lung carcinomas according to clinical variables ($n$ = 121)

| Clinical variables | Patients ($n$) | MST (weeks) | Log-rank test $P$-value | Relative risk |
|---|---|---|---|---|
| Total | 121 | 107 | – | – |
| Age | | | | |
| ≤ 58 | 66 | 92 | NS (0.51) | 1.0 |
| > 58 | 55 | 111 | | 1.2 |
| Sex | | | | |
| Male | 113 | 108 | NS (0.09) | 1.0 |
| Female | 8 | 38 | | 1.9 |
| Tumour extent | | | | |
| T 1,2 | 38 | 128 | 0.026 | 1.0 |
| T 3 | 83 | 85 | | 1.9 |
| Lymph node involvement | | | | |
| Negative | 51 | 165 | 0.024 | 1.0 |
| Positive | 70 | 68 | | 1.7 |
| Stage | | | | |
| I, II | 33 | 128 | NS (0.06) | 1.0 |
| III | 88 | 85 | | 1.7 |

NS, not significant.

**Table 2** Median survival times (MST) of patients with squamous cell lung carcinomas according to cellular factors ($n$ = 121)

| Variables | Patients ($n$) | MST (weeks) | Log-rank test $P$-value | Relative risk |
|---|---|---|---|---|
| ERBB-1 | | | | |
| Negative | 21 | > 260 | 0.018 | 1.0 |
| Positive | 100 | 92 | | 2.3 |
| VEGF | | | | |
| Negative | 97 | 117 | 0.006 | 1.0 |
| Positive | 12 | 47 | | 2.3 |
| cyclin A | | | | |
| Negative | 29 | > 260 | 0.028 | 1.0 |
| Positive | 78 | 87 | | 1.9 |
| FOS | | | | |
| Negative | 49 | > 260 | 0.009 | 1.0 |
| Positive | 72 | 92 | | 1.9 |
| JUN | | | | |
| Negative | 69 | 117 | 0.029 | 1.0 |
| Positive | 51 | 84 | | 1.6 |
| MYC | | | | |
| Negative | 62 | 102 | NS (0.9) | – |
| Positive | 59 | 112 | | – |

Tumour material was not available for all measurements (missing data: JUN one; cyclin A 14; VEGF, 12).

procedures only and 27 patients received palliative irradiation. The additional radiation treatment had no significant effect on patient survival time. Follow-up data were obtained from hospital charts and by corresponding with the referring physicians. Survival times were determined from the day of surgery. Eight patients who died within 4 weeks after surgery were excluded from the analysis.

### Immunohistochemistry

The previously described biotin–streptavidin method (Volm et al, 1988; 1993) was used to detect FOS, JUN, MYC, ERBB-1, cyclin A and VEGF. To detect the c-*fos* gene product we used the rabbit polyclonal antibody c-fos Ab-2 (Dianova, Hamburg, Germany). This antibody was developed against a peptide corresponding to residues 4–17 of human FOS. The c-*jun* gene product was detected by the rabbit polyclonal antibody c-jun/AP-1 (Ab-1, Dianova). This antibody was developed against a peptide corresponding to the amino acids 209 to 225 in the DNA binding domain of v-*jun*, C-terminal region. To detect the c-*myc* product the mouse monoclonal antibody c-myc (Ab-3, Dianova) was applied. The rabbit polyclonal antibody EGFR (Ab-4, Dianova) was used to detect the gene product of c-*erb*B-1. This antibody was raised against a peptide corresponding to the amino acid residues 1005 to 1016 of EGFR. For staining of VEGF, a rabbit polyclonal anti-VEGF antibody (Ab-2, Dianova) was used. Staining for cyclin A protein was undertaken with a rabbit polyclonal antibody (cyclin A, H-432, Santa Cruz Biotechnology, Heidelberg, Germany) that

© Cancer Research Campaign 1998

**Figure 2** Kaplan–Meier estimates of patient survival in cases of squamous cell lung carcinomas (total $n$ = 121 patients). Additional assessments are presented according to tumour extent (T), lymph node involvement (LN) and the expression of ERBB-1, VEGF, cyclin A, FOS, JUN and MYC

corresponded to amino acids 1–423 and represented full-length human cyclin A.

Formalin-fixed and paraffin-embedded tumour sections were deparaffinized according to standard histological procedures. After preincubation with hydrogen peroxide (0.3%), saponin (0.05%), unlabelled streptavidin and non-immunized normal serum (1:10, 15 min), the primary antibodies were applied for 16 h at 4°C in a moist chamber. This antibody application occurred at a concentration between 1 and 10 μg $ml^{-1}$. The most appropriate concentration was determined in preliminary experiments. After washing three times with phosphate-buffered saline (PBS), the sections were incubated for 45 min at room temperature in the presence of biotinylated goat anti-rabbit Ig or sheep anti-mouse Ig (1:50) as a secondary antibody (both with 5% normal human serum). Thereupon, the streptavidin biotinylated peroxidase complex (Amersham, Braunschweig, Germany; dilution 1:100, 15 min) was added. After washing three times with PBS and incubation in 0.5% Triton X-100 (30 s), the peroxidase activity was made visible with 3-amino-9-ethylcarbazole (15 min). Counter-staining was performed with haematoxylin. Negative controls were prepared by omitting the primary antibodies and by substituting irrelevant antibodies for the primary antibodies.

The immunohistochemical staining was analysed according to a scoring method previously validated by us in a series of animal and human cell lines and human solid tumours. Without having any previous knowledge of each patient's clinical data, three observers independently evaluated the results from the staining procedure. The evaluations agreed in 90% of the samples. The

© Cancer Research Campaign 1998

Table 3 Relationship between lymph node involvement (LN) and cellular factors

| Variables | LN-negative | LN-positive | P-value |
|---|---|---|---|
| ERBB-1 | | | |
| Negative | 10 | 11 | NS (0.57) |
| Positive | 41 | 59 | |
| VEGF | | | |
| Negative | 40 | 57 | NS (0.60) |
| Positive | 4 | 8 | |
| cyclin A | | | |
| Negative | 14 | 15 | NS (0.30) |
| Positive | 29 | 49 | |
| FOS | | | |
| Negative | 21 | 28 | NS (0.90) |
| Positive | 30 | 42 | |
| JUN | | | |
| Negative | 35 | 34 | 0.019 |
| Positive | 15 | 36 | |

Table 4 Median survival times (MST) of the subgroup of patients with squamous cell lung carcinomas and positive lymph node status ($n$ = 70) subdivided according to the expression of cellular factors

| Variables | Patients ($n$) | MST (weeks) | Log-rank test P-value | Relative risk |
|---|---|---|---|---|
| ERBB-1 | | | | |
| Negative | 11 | 43 | NS (0.61) | – |
| Positive | 59 | 69 | | |
| VEGF | | | | |
| Negative | 57 | 88 | 0.005 | 1.0 |
| Positive | 8 | 28 | | 2.8 |
| cyclin A | | | | |
| Negative | 15 | > 260 | 0.027 | 1.0 |
| Positive | 49 | 57 | | 2.5 |
| FOS | | | | |
| Negative | 28 | 128 | 0.008 | 1.0 |
| Positive | 42 | 47 | | 2.2 |

remaining specimens (10%) were independently re-evaluated and then categorized according to the classification made most frequently by the observers.

To assess the protein expression, a score corresponding to the sum of (a) the percentage of positive cells (0 = no immunopositive cells; 1 = < 25% positive cells; 2 = 26–50% positive cells; and 3 = > 50% positive cells) and (b) the staining intensity (0 = negative; 1 = weak; 2 = moderate; 3 = high) was established. Tumours were classified as negative for FOS, JUN, MYC and cyclin A when staining was completely absent and were deemed positive when the above-mentioned factors attained a cumulative score from 2 to 6 (score 1 is impossible). Tumours were graded as VEGF negative and ERBB1 negative when they yielded a score between 0 and 2 compared with the baseline value. In these cases, tumours with scores of 3 and 4 were classified as weakly positive and those with scores of 5 and 6 as strongly positive.

ERBB-1 was detected at the cell membrane (Figure 1A); VEGF was found distributed in the cytoplasm (Figure 1B); cyclin A exhibited both cytoplasmic and nuclear immunoreactivity; MYC (Figure 1C), FOS and JUN were expressed in the nucleus.

### Statistical analysis

Patient survival time was determined from the date of surgery until the last follow-up visit or reported death, and was evaluated by using life table analyses according to the method of Kaplan and Meier. Groups were compared using long-rank tests (Kalbfleisch and Prentice, 1980). The prognostic influence of clinical and molecular parameters was examined by multivariate regression methods (Cox model) (see Kalbfleisch and Prentice, 1980). A variable selection strategy was used similar to that described by Byar (1982). The correlations between clinical and molecular parameters were statistically evaluated by using Fisher's exact test (Fleiss, 1973). Throughout this paper a $P$-value of $\leq 0.05$ is considered to be statistically significant.

### RESULTS

The purpose of the analysis was to ascertain whether or not combining the expression of molecular and cellular factors with clinical prognostic factors can yield improved prognostic value for patients with squamous cell lung carcinomas.

The overall prognosis of patients with squamous cell lung carcinomas is mainly determined by tumour extent (T) and lymph node involvement (LN). This holds true for our group of patients (Table 1). Patients with T3 tumors and positive lymph nodes (LN) had significantly shorter survival times (T, $P$ = 0.026; LN, $P$ = 0.024). The median survival time for patients with T1 or T2 tumours was 128 weeks and for patients with T3 tumours, only 85 weeks. The relative risk estimate for patients with T3 tumours was 1.9 when compared with patients with T1 or T2 tumours. Patients without any lymph node involvement had median survival times of 165 weeks. With lymph node involvement this decreased to 68 weeks. The relative risk estimates for patients with a positive lymph node status was 1.7. Stage and sex exhibited only a borderline significance; age had no effect on survival.

To discover new cellular prognostic factors in addition to tumour extent and lymph node involvement, the expression of several molecular and cellular factors was analysed (Table 2). Of the 121 squamous cell lung carcinomas, 21 (17%) did not show ERBB-1 expression, whereas 100 (83%) showed positive staining. The median survival was shorter for patients with ERBB-1-positive tumours than for those with ERBB1-negative tumours (92 vs > 260 weeks; $P$ = 0.018). The relative risk estimate for those patients was increased by a factor of 2.3. High VEGF expression was found in 12 instances (11%), whereas 97 cases (89%) did not express VEGF or demonstrated only moderate expression. Patients with VEGF-positive tumour cells had significantly lower median survival times (47 vs 117 weeks) than patients with negative or only moderately stained cells ($P$ = 0.006). The survival times were significantly shorter in patients with cyclin A-positive tumours than in patients with cyclin A-negative tumours (87 vs > 260 weeks, $P$ = 0.028).

A total of 72 out of 121 (60%) squamous cell lung carcinomas were positive for FOS; 51 out of 120 (43%) were positive for JUN; 59 out of 121 (49%) were positive for MYC. Median survival times were significantly shorter for patients with FOS positive (92 vs > 260 weeks; $P$ = 0.009) and with JUN positive (84 vs 117 weeks; $P$ = 0.029) carcinomas (Table 2). The expression of MYC in squamous cell lung carcinomas showed no significant correlation with survival. Furthermore, other assorted parameters (e.g. RAS, ERBB-2) had no significant correlation with survival (data

© Cancer Research Campaign 1998

not shown). Figure 2 shows the survival curves (Kaplan–Meier estimates) of patients forming subgroups according to clinical and cellular factors.

A significant relationship between tumour extent (T) and the factors ERBB-1, VEGF, cyclin A, FOS and JUN was not observed (data not shown). ERBB-1, VEGF, cyclin A and FOS also acted independently of lymph node involvement for the patient population examined here (Table 3). However, JUN was expressed to a significantly higher frequency in patients with positive lymph node involvement ($P = 0.019$; Table 3).

The factors ERBB-1, VEGF, cyclin A and JUN presented no inter-relationships or interdependencies. Only the presence of FOS was strongly correlated with ERBB-1 ($P = 0.001$), VEGF ($P = 0.02$), and JUN ($P = 0.0009$) being positive.

To ascertain whether the expression of ERBB-1, VEGF, cyclin A and FOS can add prognostic information to the important clinical factors, we determined the median survival of patients with positive lymph node involvement and the corresponding dependence upon the expression or non-expression of these factors (Table 4). In this analysis, survival times were also shorter in the VEGF-positive, cyclin A-positive and FOS-positive groups of patients. For patients with VEGF-negative carcinomas the median survival time was 88 weeks and for patients with VEGF-positive carcinomas only 28 weeks ($P = 0.005$). The corresponding values for cyclin A and FOS were > 260 vs 57 weeks ($P = 0.027$) and 128 vs 47 weeks ($P = 0.008$) respectively. Figure 3 shows the survival curves (Kaplan–Meier estimates) for patients with positive lymph node status according to the expression of VEGF, cyclin A and FOS. It can clearly be seen that the prognostic value can be improved by combining clinical and cellular or molecular parameters.

In addition to descriptively evaluating the prognostic relevance of the identified clinical and cellular factors as mentioned above, we also analysed the data from the 121 squamous cell carcinoma patients using the proportional hazards regression model (Cox model). The factors were introduced into a multivariate regression analysis that consisted of several consecutive steps as follows: lymph node involvement and tumour extent were significant prognostic clinical factors obtained from the univariate analysis. As both factors were highly correlated, only one factor could be simultaneously included in a multivariate analysis. Reported next are the results obtained using lymph node involvement as the only classical factor (negative vs positive). Based upon the immunohistochemistry parameters, we had to consider VEGF, ERBB-1, FOS and cyclin A as possible candidates for a multivariate analysis, because they had shown a significant prognostic value in the univariate analysis. JUN was not included because of its positive correlation with lymph node involvement and other cellular parameters such as FOS. Next, we investigated separately VEGF, ERBB1, FOS and cyclin A for their prognostic value when combined with lymph node involvement (LN). Table 5 shows that VEGF and FOS demonstrated the strongest prognostic influence ($P = 0.003$ and $P = 0.007$) independent of LN, whereas, the effect of ERBB-1 and cyclin A was just barely significant ($P = 0.04$). When comparing the relative risk of patients with positive lymph nodes (LN) or patients with positive cellular parameters, we found a relative risk over baseline between 1.6 and 2.6. It was highest in the analysis of LN together combined with VEGF (2.0 and 2.6). The next highest risks were observed in the analysis of LN together with FOS (1.8 and 2.0). This indicates that good prognostic differentiation can be achieved by using LN combined with VEGF. To a somewhat smaller extent, it is also possible to use LN and FOS. The parameters ERBB-1 and cyclin A yielded a lower prognostic value.

**Table 5** Bivariate analysis of the prognostic value exhibited by the cellular parameters that had shown an effect upon the survival time during univariate analysis (Table 2) and lymph node involvement (LN)

| | Beta | RR | *P*-value |
|---|---|---|---|
| LN | 0.69 | 2.0 | 0.008 |
| VEGF | 0.95 | 2.6 | 0.003 |
| LN | 0.47 | 1.6 | 0.05 |
| ERBB-1 | 0.79 | 2.2 | 0.04 |
| LN | 0.57 | 1.8 | 0.02 |
| FOS | 0.67 | 2.0 | 0.007 |
| LN | 0.48 | 1.6 | 0.06 |
| cyclin A | 0.64 | 1.9 | 0.04 |

RR, relative risk.

**Figure 3** Kaplan–Meier estimates of patient survival in individuals having squamous cell lung carcinomas accompanied by a positive lymph node status and according to the expression of VEGF, cyclin A and FOS

© Cancer Research Campaign 1998

Table 6 Multivariate analysis of the prognostic value of all five factors (a) and of the two most important ones (LN and VEGF) combined with one of the remaining factors for the survival time (b–d)

| | | Beta | RR | P-value |
|---|---|---|---|---|
| (a) | LN | 0.65 | 1.9 | 0.02 |
| | VEGF | 0.64 | 1.9 | 0.04 |
| | FOS | 0.40 | 1.5 | 0.17 |
| | cyclin A | 0.37 | 1.5 | 0.28 |
| | ERBB-1 | 0.23 | 1.3 | 0.47 |
| (b) | LN | 0.71 | 2.0 | 0.006 |
| | VEGF | 0.77 | 2.2 | 0.02 |
| | FOS | 0.61 | 1.8 | 0.03 |
| (c) | LN | 0.65 | 1.9 | 0.02 |
| | VEGF | 0.80 | 2.2 | 0.02 |
| | cyclin A | 0.45 | 1.6 | 0.18 |
| (d) | LN | 0.66 | 1.9 | 0.01 |
| | VEGF | 0.80 | 2.2 | 0.02 |
| | ERBB1 | 0.51 | 1.7 | 0.09 |

RR, relative risk.

Table 7 Multivariate analysis of the prognostic value of tumour size as described by T (a) or stage (b) and the cellular parameters for the overall survival

| | | Beta | RR | P-value |
|---|---|---|---|---|
| (a) | T | 0.49 | 1.6 | 0.08 |
| | VEGF | 0.60 | 1.8 | 0.07 |
| | FOS | 0.52 | 1.7 | 0.06 |
| (b) | Stage | 0.45 | 1.6 | 0.13 |
| | VEGF | 0.58 | 1.8 | 0.08 |
| | FOS | 0.57 | 1.8 | 0.04 |

RR, relative risk.

A multivariate analysis of all five factors indicated that LN and VEGF were the most important factors ($P$ = 0.02 and $P$ = 0.08, Table 6a). The relative risk estimates of positive LN and positive VEGF were 1.9. When we simplified the multivariate model and only used LN, VEGF and FOS, all three parameters showed a significant prognostic value (Table 6b). When we analysed LN, VEGF, and cyclin A (Table 6c) or LN, VEGF and ERBB-1 only LN and VEGF were significant (Table 6d).

Next we examined separately the prognostic influence of the cellular parameters in the two LN strata (positive or negative). None of the four cellular parameters demonstrated any significant influence in the subgroup of patients ($n$ = 38) that could be evaluated and that had negative LN in the multivariate analysis ($P$ > 0.2). In the subgroup of patients ($n$ = 68) that could be evaluated and that had positive nodes, VEGF was the sole significant factor ($P$ = 0.04). FOS tended to exert some influence ($P$ = 0.13). When using VEGF as the only factor to explain the variation of the survival times, it was clearly significant ($P$ = 0.02) in that subgroup and yielded a relative risk estimate of 2.7. These findings were confirmed by a Cox model using LN as the stratum variable. In this instance, VEGF was the only significant factor ($P$ = 0.02); it resulted in a relative risk estimate of 2.3 using a backward selection procedure. Furthermore, the all subset selection procedure identified LN as the most important single factor, LN and VEGF were deemed the most important pair of factors, and LN, VEGF and FOS were the most important triple of factors.

Returning to the selection of the clinical variables in which LN had been clearly selected as the most significant factor, we also investigated whether or not the selection of cellular factors could have been influenced by the choice of clinical factors. Therefore, we analysed VEGF and FOS along with tumour extent and tumour stage (Table 7). The results confirmed that VEGF and FOS are important prognostic factors for survival of patients with squamous cell lung carcinomas.

### DISCUSSION

This investigation attempted to prove the prognostic value of several cellular factors, measured using immunohistochemistry, of patients with squamous cell lung cancer. This study clearly shows that immunohistochemical analysis of cellular factors possesses independent prognostic significance for patients with squamous cell lung carcinomas.

As the oncoproteins FOS and JUN co-operate in the activation of transcription from specific promoter elements, it is conceivable that they might also co-operate in inducing a proliferative state (Auwerx and Sassone-Corsi, 1991). In our analysis, we did indeed find that (a) FOS and JUN expression is highly correlated and that (b) patients with FOS- and JUN-positive carcinomas had a poorer clinical outcome than other patients with squamous cell lung carcinomas. The c-*erb*B-1 gene product is also involved in the regulation of cell growth and proliferation (Carney, 1991). In our investigation we discovered that patients with an expression of ERBB-1 had shorter survival times. Spandidos et al (1990) examined MYC expression in specimens of bronchogenic carcinomas by immunohistochemical means and found that this protooncogene product is important for tumour progression. However, we could detect no correlation between elevated MYC and patient survival. Earlier research conducted by us found that MYC was more frequently expressed in lymph node metastases of patients with primary lung carcinomas (Volm et al, 1994). In the present investigation, this result could be confirmed for squamous cell lung carcinomas. However, we could also show that MYC is not a prognostic indicator for the survival time of patients with squamous cell lung carcinomas. Other investigated factors (e.g. RAS, ERBB-2) did not show any association with survival (data not shown).

Along with other researchers (Dutta et al, 1995; Paterlini et al, 1995), we found that the expression of cyclin A is closely correlated to the proportion of S-phase cells as measured by flow cytometry (data not shown). Univariate analysis indicates that cyclin A may also be a good prognostic indicator for the survival of patients with squamous cell lung carcinomas, but this was no longer seen in the multivariate analysis.

Tumour angiogenesis is thought to be mediated by different factors. One such factor is VEGF, a dimeric glycoprotein. $VEGF_{165}$, which we analysed, is the most abundant isoform (Ferrara et al, 1992). Toi et al (1994) found that the relapse-free survival rate of patients with VEGF-rich breast carcinomas was significantly worse than that of patients with VEGF-poor tumours. Similarly, Maeda et al (1996) noted a significantly shorter survival in patients with VEGF-positive gastric carcinomas than in those having VEGF-negative tumours. Those data and our own indicate that VEGF expression may be an additional prognostic indicator.

© Cancer Research Campaign 1998

In addition to evaluating the prognostic relevance of the cellular factors descriptively, we analysed the data using the proportional hazards regression model (Cox model). In our analysis, VEGF and FOS revealed the strongest prognostic influence on survival, independent of lymph node involvement. The other cellular factors (ERBB-1 and cyclin A) were only of borderline significance.

In addition, we examined whether the selection of the cellular factors could have been influenced by the choice of clinical parameters. Therefore, we also analysed the cellular factors along with tumour extent and tumour stage. The results with tumour extent and tumour stage confirmed the data obtained with lymph node involvement.

In this study, we found that in addition to the very important factor of lymph node involvement other cellular parameters (VEGF, FOS) also serve as prognostic factors of the patient's clinical outcome in squamous cell lung carcinomas. These supplemental prognostic factors are readily available at an acceptable cost.

### REFERENCES

Auwerx J and Sassone-Corsi P (1991) IP-1: a dominant inhibitor of FOS/JUN whose activity is modulated by phosphorylation. *Cell* **64**: 983–993

Byar DR (1982) Analysis of survival data. Cox and Weibull models with covariates. In *Statistics in Medical Research. Methods and Issues with Application in Cancer Research*, Mike V and Stanley KE (eds), pp. 365–401. J Wiley: New York

Carney WP (1991) Oncogenes and non-oncogene related human tumor antigens. *Cancer J* **4**: 156–161

Carr DT and Mountain CF (1977) Staging lung cancer. In *Lung Cancer. Clinical Diagnosis and Treatment*, Strauss MJ (ed.), pp. 151–161. Grune & Stratton: New York

Cordon-Cardo C (1995) Mutation of cell cycle regulators. Biological and clinical implications for human neoplasia (review). *Am J Path* **147**: 545–560

Dutta A, Chandra R, Leiter L and Lester S (1995) Cyclins as markers of tumor proliferation: Immunocytochemical studies in breast cancer. *Proc Natl Acad Sci USA* **92**: 5386–5390

Ferrara N, Houck K, Jakeman L and Leung DW (1992) Molecular and biological properties of the vascular endothelial-growth-factor family of proteins. *Endocrine Rev* **13**: 8–32

Fleiss JL (1973) *Statistical Methods for Rates and Proportion.* J Wiley: New York

Kalbfleisch I and Prentice L (1980) *Analysis of Failure Time Data.* J Wiley: New York

Maeda K, Chung YS, Ogawa Y, Takatsuka S, Kang S-M, Ogawa M, Sawada T and Sowa M (1996) Prognostic value of vascular endothelial growth factor expression in gastric carcinoma. *Cancer* **77**: 858–863

Moolenaar WH, Rob JA, Tertoolen LGJ and de Laat SW (1986) The epidermal growth factor induced signal in A 431 cells. *J Biol Chem* **261**: 279–284

Paterlini P, Flejou JF, de Mitri MS, Pisi E, Franco D and Brechot C (1995) Structure and expression of the cyclin A gene in human primary liver cancer. Correlation with low cytometric parameters. *J Hepatol* **23**: 47–52

Sassone-Corsi P, Lamph WW, Kamps M and Verma IM (1988) FOS-associated cellular p39 is related to nuclear transcription factor AP-1. *Cell* **54**: 553–563

Spandidos DA, Zakinthinos S, Petraki C, Sotsiou F, Yiagnisis M, Dimopoulos AM, Roussos G and Field JK (1990) Expression of ras p21 and myc p62 oncoproteins in small cell and non-small cell carcinomas of the lung. *Anticancer Res* **10**: 1105–1114

Toi M, Hoshina S, Takayanagi T and Tominaga T (1994) Association of vascular endothelial growth factor expression with tumor angiogenesis and with early relapse in primary breast cancer. *Jpn J Cancer Res* **85**: 1045–1049

Volm M, Bak M, Efferth T, Lathan B and Mattern J (1988) Immunocytochemical detection of a resistance-associated glycoprotein in tissue culture cells, ascites tumors and human tumor xenografts by Mab 265/F4. *Anticancer Res* **8**: 531–536

Volm M, Mattern J and Samsel B (1993) Relationship of inherent resistance to doxorubicin, proliferative activity and expression of P-glycoprotein, and glutathione S-transferase-$\pi$ in human lung tumors. *Cancer* **71**: 3181–3187

Volm M, van Kaick G and Mattern J (1994) Analysis of c-fos, c-jun, c-erbB1, c-erbB2 and c-myc in primary lung carcinomas and their lymph node metastases. *Clin Exp Metast* **12**: 329–334

Weidner N (1995) Intratumorous microvessel density as a prognostic factor in cancer. *Am J Path* **147**: 9–19

World Health Organization (1981) Histological typing of lung tumors. *Tumori* **6**: 253–272

© Cancer Research Campaign 1998