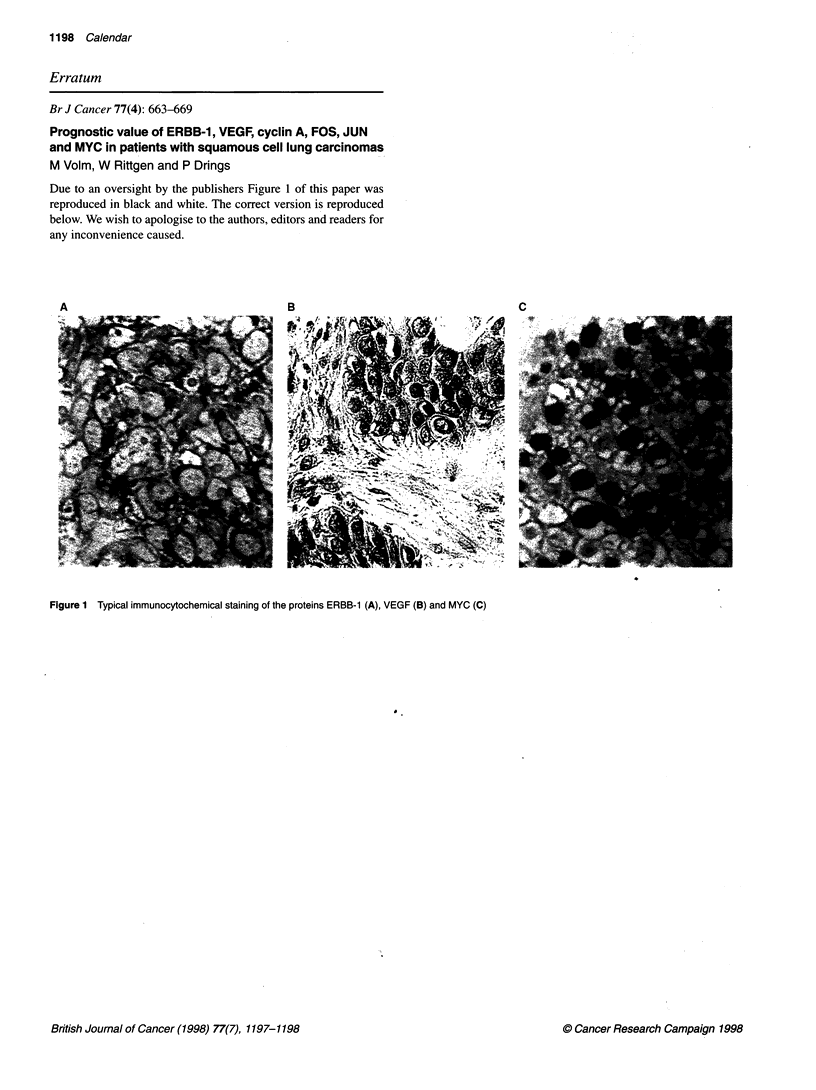# Prognostic value of ERBB-1, VEGF, cyclin A, FOS, JUN and MYC in patients with squamous cell lung carcinomas

**Published:** 1998-04

**Authors:** 

## Abstract

**Images:**


### *Erratum*

*Br J Cancer* 77(4): 663–669

**Prognostic value of ERBB-1, VEGF, cyclin A, FOS, JUN and MYC in patients with squamous cell lung carcinomas**

M Volm, W Rittgen and P Drings

Due to an oversight by the publishers Figure 1 of this paper was reproduced in black and white. The correct version is reproduced below. We wish to apologise to the authors, editors and readers for any inconvenience caused.

**Figure 1** Typical immunocytochemical staining of the proteins ERBB-1 (**A**), VEGF (**B**) and MYC (**C**)

© *Cancer Research Campaign 1998*